# A new volar plate made of carbon-fiber-reinforced polyetheretherketon for distal radius fracture: analysis of 40 cases

**DOI:** 10.1007/s10195-014-0311-1

**Published:** 2014-07-15

**Authors:** Luigi Tarallo, Raffaele Mugnai, Roberto Adani, Francesco Zambianchi, Fabio Catani

**Affiliations:** 1 Orthopaedics and Traumatology Department, Modena Policlinic, University of Modena and Reggio Emilia, Via del Pozzo 71, 41124 Modena, Italy; 2 Department of Hand Surgery and Microsurgery, University Hospital of Verona, Verona, Italy

**Keywords:** DiPHOS, Plate, Radius, Fracture, PEEK, Radiolucent, Carbon-fiber-reinforced

## Abstract

**Background:**

Implants based on the polyetheretherketon (PEEK) polymer have been developed in the last decade as an alternative to conventional metallic devices. PEEK devices may provide several advantages over the use of conventional orthopedic materials, including the lack of metal allergies, radiolucency, low artifacts on magnetic resonance imaging scans and the possibility of tailoring mechanical properties. The purpose of this study was to evaluate the clinical results at 12-month follow-up using a new plate made of carbon-fiber-reinforced polyetheretherketon for the treatment of distal radius fractures.

**Materials and methods:**

We included 40 consecutive fractures of AO types B and C that remained displaced after an initial attempt at reduction. The fractures were classified according to the AO classification: 21 fractures were type C1, 9 were type C2, 2 were type C3, 2 were type B1 and 6 were type B2.

**Results:**

At a 12-month follow-up no cases of hardware breakage or loss of the surgically achieved fracture reduction were documented. All fractures healed, and radiographic union was observed at an average of 6 weeks. The final Disabilities of Arm, Shoulder and Hand score was 6.0 points. The average grip strength, expressed as a percentage of the contralateral limb, was 92 %. Hardware removal was performed only in one case, for the occurrence of extensor tenosynovitis.

**Conclusion:**

At early follow-up this device showed good clinical results and allowed maintenance of reduction in complex, AO fractures.

**Type of study/level of evidence:**

Therapeutic IV.

## Introduction

Open reduction and internal fixation using pre-contoured plates has become a surgical treatment option for displaced, unstable and comminuted fractures of the distal radius. They provide immediate stable fixation allowing early mobilization, which can result in rapid recovery and improved regain of function [1, 2]. Fixed angle plates using locking-screw technology allow surgeons to manage complex periarticular fractures since they give distal stability by direct support of the subchondral bone and do not depend on distal screw purchase to maintain reduction [1].

Distal radius plating can be performed using a dorsal or volar approach; however, a higher rate of tendon irritation and rupture has been reported with the use of dorsal plates [3]. Implants based on the polyetheretherketon (PEEK) polymer have been developed in the last decade as an alternative to conventional metallic devices. PEEK devices may provide several advantages over the use of conventional orthopedic materials, including the lack of metal allergies, radiolucency, low artifact interference on magnetic resonance imaging scans and the possibility of tailoring mechanical properties [4]. In fact, compared with clinically used metallic implants, CFR-PEEK implants can be designed with more appropriate strength, toughness, or stiffness by the arrangement of reinforcing fiber volume and orientation, and can provide better fatigue resistance [5].

Although neat (unfilled) PEEK biomaterials can exhibit an elastic modulus ranging between 3 and 4 GPa, the modulus can be tailored to closely match cortical bone (18 GPa) or titanium alloy (110 GPa) by preparing carbon-fiber-reinforced (CFR) composites with varying fiber length and orientation [6]. Therefore, PEEK has a more similar stiffness to bone than titanium.

A recent study compared the CFR-PEEK dynamic compression plate, distal radius volar plate, proximal humeral plate, and tibial nail to commercially available devices regarding the biomechanical characteristics (by four-point bending, static torsion of the nail, and bending fatigue) and the wear/debris (by amount of the debris generated at the connection between the CFR-PEEK plate and titanium alloy screws). The authors concluded that CFR-PEEK and metal implants yielded similar biomechanical characteristics to other commercially available devices. In particular, the distal volar plate bending structural stiffness of the CFR-PEEK distal volar plate was 0.542 versus 0.376 N m^2^ for the DePuy’s DVR anatomic volar plate. All tested CFR-PEEK devices underwent one million fatigue cycles without failure. Moreover, the wear test showed that the accumulated debris on the 1 mm filters weighed very little, i.e. an average of 0.78 mg of the CF-PEEK material in comparison to 5.35 mg of the titanium sample [7].

Numerous studies documenting the successful clinical performance of CFR-PEEK in orthopedic, trauma and spinal surgery continue to emerge in the literature [8–10].

The research presented here represents the continuation of a previously reported study [11] with the aim to evaluate the clinical results at a 12-month follow-up, using the new DiPHOS-RM plate made of CFR-PEEK for the treatment of the distal radius fracture.

## Materials and methods

We performed a prospective study including all patients who were treated for unstable distal radius fracture with a volar fixed angle plate DiPHOS-RM produced by Lima Corporate (Villanova di San Daniele Del Friuli, Udine, Italy), during a period of 7 months (between March 2012 and September 2012). We included all the consecutive fractures of AO types B and C that remained displaced after an initial attempt at reduction. Fractures of AO type A were not included since they were treated nonsurgically. The patients were 16 men and 24 women with an average age of 65 years at the time of injury (range 26–82). The mechanisms of injury were simple falls on outstretched hands in 22 cases, motor vehicle accidents in 7 cases and sports injuries in another 11 cases. The fractures were classified according to the AO classification: 21 fractures were type C1, 9 were type C2, 2 were type C3, 2 were type B1 and 6 were type B2.

A preoperative computed tomography scan was carried out on all patients affected by type C fractures and in one patient a postoperative computed tomography scan was also obtained.

**Fig. 1:**
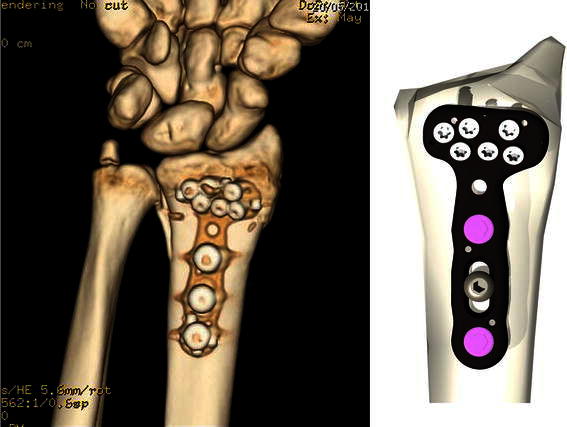
3-D reconstruction of the new DiPHOS-RM plate and CT scan in 3-D showing an implanted plate in a C1-type fracture

In this study, the researched plate (DiPHOS-RM, Lima corporate, Villanova di San Daniele, Italy) was manufactured by injection molding of CFR-PEEK and consists of two distal rows of holes for the 2.3 locking screws on the distal part of the plate, and three or more different holes for 3.5 locked screws on the diaphysis (Fig. 1).

The main differences between a CFR-PEEK plate and the most common materials in trauma implants (i.e. titanium plate) are the following:

- The CFR-PEEK is completely radiolucent, a property that allows direct evaluation of osseous callus formation because consolidation and mineralization is not obscured by the plate in both standard views. The position of the plate can be detected anyway in the radiographic controls thanks to tantalum markers positioned on the distal and proximal borders.
- The specific design of the holes of the CFR-PEEK plate and of the head of the screw allows the insertion of the screws in multiple directions with high strength coupling.
- The threaded coupling between the head of the screw and the plate holes in the titanium angular stability plates could cause cold welding, while coupling of screws and plate of different materials precludes cold welding.
- The elastic modulus of CFR-PEEK is similar to the modulus of the cortical bone, an advantageous feature for an osteosynthesis device, in order to prevent reduction of bone quality adjacent to the plate [12].

In order to reach a strong angular stability between plate and screws, the right choice of material and production process (injection molding) combined with a specific geometry of the holes of the plate and of the thread of the head of the screws was made.

The self-threading screws were inserted using the specific screwdriver according to the surgical technique in order to completely insert the screw into the hole of the plate.

**Fig. 2:**
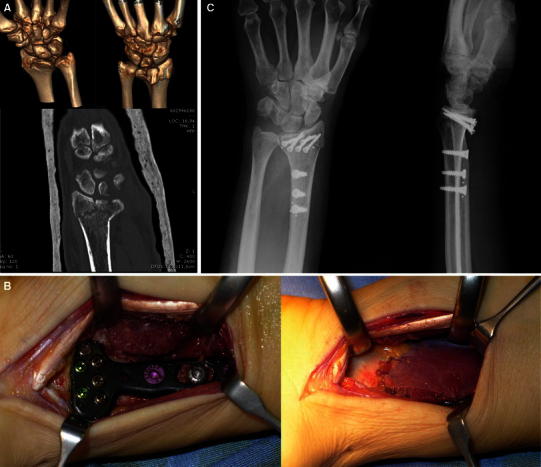
**a** Preoperative 2- and 3-D CT scan showing a C1-type wrist fracture according to the AO classification. **b** Intraoperative view of distal radius fracture with the DiPHOS-RM implanted and the pronator quadratus re-attached. **c** X-ray evaluation performed 3 months after surgery showing good healing of the fracture

The operations were performed using Henry’s volar approach. The plate was placed directly on the radius after the reduction of the fracture, and the adequate positioning of plate and screws were confirmed by intraoperative fluoroscopy. Finally, the square pronator was sutured, allowing an almost complete coverage of the plate (Fig. 2).

A below-elbow wrist splint was used for 2 weeks in all cases. At the end of the 2nd week, the sutures were removed and physiotherapy started.

As we usually do in our clinical practice, all patients were clinically and radiologically revised at 1, 2, and 3 months, and thereafter at 6-monthly intervals.

The clinical and radiological evaluation was performed by one of the authors and included analysis of passive range of motion (ROM), grip strength, functional outcome, radiological evaluation of fracture healing and reduction maintenance.

The X-ray assessment included standard antero-posterior and lateral projections of the injured wrist, and reduction maintenance was determined by assessing radial inclination, tilt, ulnar variance, step off, and gap. All measurements were performed on a picture archiving and communication system (PACS, software Fuji Synapse).

Time of union was determined according to both radiological and clinical parameters. Radiological criteria included: bridging of the fracture site by bone, callus or trabeculae; bridging of the fracture seen at the cortices; and obliteration of the fracture line or cortical continuity. Clinical criteria were represented by the patient’s ability to bear weight on the injured limb and perform activities of daily living, and the presence of pain at the fracture site upon palpation and physical stress.

Clinical results were assessed with physician-directed outcome tools and with subjective questionnaires after surgery.

Grip strength was measured with a Jamar dynamometer (Asimov Engineering Corp, Santa Monica, CA), and wrist ROM using a goniometer. Functional outcome was performed with the Disabilities of Arm, Shoulder and Hand (DASH) questionnaire. This instrument quantifies disabilities related to the upper extremity with a score ranging from 0 points (no disability) to 100 points (maximum disability) [13]. Possible early or late complications were assessed and recorded at each follow-up evaluation.

## Results

At a 12-month follow-up all the patients included were clinically and radiologically reviewed.

Thirty-four patients who were in employment at the time of injury were able to return to work within 14 weeks of injury. All fractures healed, and radiographic union was observed at an average of 6 weeks (range 4–8 weeks). No cases of loss of the surgically achieved fracture reduction were documented. The clinical evaluation and outcome scores data at the final follow-up are reported in Table 1. In particular, the average wrist range of motion was: 65° in flexion (range 45°–80°), 55° in extension (range 40°–65°), 21.5° in radial deviation (range 5°–35°), 33.5° in ulnar deviation (range 30°–45°), 75° in supination (range 65°–90°), and 79° in pronation (range 60°–90°). The final DASH score was 6 points (range 3–16). The average grip strength, expressed as percentage of respective contralateral limb, was 92 %. No cases of hardware failure, loss of position or alignment of fixed-angle locking screws, nervous complications, infection or allergy to the plate were observed in our cohort of patients. In one case, a 55-year-old male, clinical signs of extensor tendons synovitis were reported 6 months after surgery. The diagnosis of extensor tenosynovitis was primarily based on the symptoms of pain, swelling, tenderness, and dorsal crepitus. Radiographs revealed an excessive length of one screw of the distal branch of the plate, after which the plate and the screws were removed. Intraoperative hardware osseointegration was found to be limited, facilitating, therefore, removal of the plate.

**Table 1:** Demographic characteristics, fracture type, clinical outcome and specific complications

Sex	Age	Fracture type/side	ROM					
			Flexion (°)	Extension (°)	Supination (°)	Pronation (°)	Grip strength (%)^a^	Complications
F	85	B2/left	80	60	70	85	90	None
F	78	C1/right	45	45	80	80	80	None
F	80	C1/left	50	60	75	80	100	None
F	76	C1/right	45	55	65	70	90	None
M	67	C1/right	75	50	65	75	80	None
F	75	C2/right	60	60	80	85	100	None
F	26	C1/right	55	50	85	90	65	None
M	57	C1/left	70	45	75	80	95	None
F	63	C3/left	50	50	65	75	95	None
F	66	C2/right	55	55	70	80	100	None
F	82	C1/right	65	55	65	75	80	None
F	74	C1/left	60	65	70	75	90	None
F	72	C1/left	70	60	85	80	95	None
F	58	C1/left	65	60	70	85	100	None
F	64	C1/right	75	50	75	85	85	None
F	63	C2/right	60	45	65	70	80	None
F	33	B2/left	60	50	80	75	100	None
M	55	C1/left	80	60	90	75	100	Extensor tenosynovitis
M	59	B2/left	65	55	70	70	100	None
F	59	C1/left	80	65	75	80	100	None
F	65	C1/left	60	45	65	78	100	None
F	64	B1/left	70	60	70	80	80	None
F	63	C2/left	65	65	70	75	75	None
M	63	B2/left	75	55	70	90	100	None
M	60	C2/right	55	50	90	85	95	None
M	77	C1/left	60	45	75	85	100	None
M	57	C2/right	80	65	80	90	100	None
M	60	C1/left	70	50	75	85	100	None
F	63	C1/right	65	50	85	90	70	None
M	72	C2/right	70	60	70	60	95	None
M	68	C1/left	80	60	75	85	95	None
M	75	C3/left	65	55	70	80	80	None
F	58	C1/right	70	60	90	75	100	None
M	82	C2/right	55	55	80	75	100	None
F	77	C1/right	80	60	65	70	95	None
M	56	C2/right	65	50	70	75	90	None
M	80	B2/left	50	50	80	90	100	None
F	54	C1/left	80	60	90	85	85	None
F	63	B2/right	55	50	75	70	85	None
M	59	B1/left	75	55	75	80	100	None

%	Mean (SD)	%	Mean (SD)	Mean (SD)	Mean (SD)	Mean (SD)	Mean (SD)	%
60 % F 40 % M	65 (12)	5 % B1 15 % B2 52 % C1 23 % C2 5 % C3	65.2 (10.4)	54.9 (6.1)	74.9 (7.7)	79.3 (6.9)	91.7 (9.7)	2.5

^a^Percentage obtained at final evaluation when compared with contralateral side

## Discussion

The primary aim in the management of unstable distal radius fractures is to obtain restoration of bony anatomy with stable internal fixation [14]. Secondary to its ability to provide stable internal fixation of a distal radius fracture, volar locking plate technology has gained significant popularity [15].

Numerous studies have reported outcomes in the good to excellent range on patient-rated scoring systems and with a relatively low rate of complications using volar plating as a treatment for unstable distal radius fractures [16–21].

In the present study the overall clinical results obtained with the use of the new DiPHOS-RM plate at 12-month follow-up are consistent with the recent literature findings using conventional metal plates [16–21].

However, the CFR-PEEK structure of the DiPHOS-RM plate has potential advantages that may support its introduction into clinical practice.

Compared with traditional implants, such as stainless steel, Co–Cr–Mo, and Ti6A14 V alloy stainless steel or titanium plates, PEEK polymer has a modulus and strengths similar to normal bone, avoiding the strong rigidity property of titanium or stainless steel plates [12, 22].

Typically, metals used in orthopedic surgery have a large elastic modulus (approximately 6–20 times greater than that of the surrounding bone) [23–25], causing impaired load force transmission at the implant–tissue interface.

Thus, according to Wolff’s law, the device may sustain far higher stresses than the bone to which it is rigidly fixed, thereby shielding the bone for stresses. Because bone requires the stimulus of mechanical stress to maintain its structure, the bone adjacent to the high modulus device becomes porotic and weaker [26, 27].

Another advantage of using a PEEK plate with metal screws is that the potential phenomenon of cold welding is eliminated. Moreover, this new plate allows in its distal part both the insertion of fixed-angle screws or distally locked screws at variable angles and with an angular range of 15° by deciding whether or not to use the provided guide. Finally, the major advantage of the DiPHOS-RM plate is its radiolucency. This characteristic allows direct visualization of osseous callus formation, allowing monitoring of the healing of the fracture, thereby improving clinical assessment and accuracy.

Therefore, specific indications for this new radiolucent plate can be represented by fractures with significant metaphyseal comminution and in cases of nascent malunion where a distal radius osteotomy with bone grafting is usually performed to correct the wrong angle.

In our cohort of patients we haven’t found any complications related to the new material of the implant, but particular care and attention is necessary while inserting the screws, as the holes of the plate are not threaded. The penetration of the screw into the hole of the plate creates a thread, allowing locking of the screws, but no more than three changes of angle is possible before the thread is ruined.

In our cohort of patients, no cases of hardware breakage, loss of the surgically achieved fracture reduction, or allergy to the plate were documented at a 12-month follow-up. The hardware was removed only in one case (5 %) for the occurrence of extensor tenosynovitis after a screw penetrated the dorsal radius cortex.

In this research we observed a limited osseointegration of this hardware, probably allowing, when necessary, an easier removal of the plate with respect to the other routinely used materials.

Compared with stainless steel or titanium plates, the cost of production of this implant is higher but the commercial price is in line with that of the metal systems.

Our first experience of using the new DiPHOS-RM plate seems favorable: in fact the plate proved to be a reliable method with good clinical and functional results at a 12-month follow-up. In addition, its distal double-row screw system and its far positioning within the distal radius epiphysis resulted in a reliable support for the articular surface, allowing maintenance of reduction even in cases of comminuted intra-articular fractures (i.e. C-type).

Future studies with a larger sample size are needed to evaluate the long-term clinical results and occurrence of possible complications at a longer follow-up using this new CFR-PEEK plate.
